# Roots of forbs sense climate fluctuations in the semi-arid Loess Plateau: Herb-chronology based analysis

**DOI:** 10.1038/srep28435

**Published:** 2016-06-21

**Authors:** Songlin Shi, Zongshan Li, Hao Wang, Georg von Arx, Yihe Lü, Xing Wu, Xiaochun Wang, Guohua Liu, Bojie Fu

**Affiliations:** 1 State Key Laboratory of Urban and Regional Ecology, Research Center for Eco-Environmental Sciences, Chinese Academy of Sciences, Beijing 100085, China; 2 University of the Chinese Academy of Sciences, Beijing 100049, China; 3 Swiss Federal Institute for Forest, Snow and Landscape Research WSL, Birmensdorf 8903, Switzerland; 4 College of Forestry, Northeast Forestry University, Harbin 150040, China

## Abstract

Growth of herbaceous plants responds sensitively and rapidly to climate variability. Yet, little is known regarding how climate warming influences the growth of herbaceous plants, particularly in semi-arid sites. This contrasts with widely reported tree growth decline and even mortality in response to severe water deficits due to climate warming around the world. Here, we use the relatively novel approach of herb-chronology to analyze the correlation between climatic factors and annual ring width in the root xylem of two perennial forb species (*Medicago sativa, Potentilla chinensis*) in the Loess Plateau of China. We show that warming-induced water deficit has a significant negative effect on the growth of herbaceous plants in the Loess Plateau. Our results indicate that the growth of forbs responds rapidly and sensitively to drought variability, implying that water availability plays a dominant role in regulating the growth of herbaceous plants in semi-arid areas. If warming and drying in the Loess Plateau continue in the future, further affects the growth of herbaceous plants, potentially driving regional changes in the relationship between herbaceous vegetation and climate.

The growth of vegetation worldwide has been strongly affected by anthropogenic climatic change in recent decades^1^,^2^,^3^,^4^, and can induce positive and negative feedbacks on climate depending on the ecosystem^5^,^6^,^7^,^8^,^9^. Recent studies have reported changing trends in vegetation as a result of recent climate change at local^10^,^11^, regional^12^,^13^, and global^4^,^14^,^15^ scales. The detection, evaluation and attribution of these trends was mainly based on observations of satellite-derived data on vegetation change, including the Normalized Difference Vegetation Index (NDVI) and Leaf Area Index (LAI)^16^,^17^. However, without corresponding ground-based measurements, it is difficult to attribute vegetation growth trends observed by satellites at the similar temporal and spatial scales^4^, and thus may fail to explain the mechanisms for the observed changes. Hence, a better understanding of the underlying drivers and mechanisms behind these trends is essential to predict how vegetation could change in response to future global change.

Many studies have reported a significant negative impact of climate change on vegetation growth as a result of water deficit driven by rapid climate warming over recent decades^4^,^12^,^17^. Furthermore, recent studies based on tree ring records have also indicated that water deficits associated with increased temperature and/or decreased precipitation, have accelerated tree growth decline^7^,^18^,^19^, or even increased tree mortality in semi-arid forests over recent decades^20^,^21^,^22^,^23^. However, there is not much known about growth responses of herbaceous vegetation to drought stress in semi-arid areas.

The recently developed ‘herb-chronology’, that is, the analysis of annual growth rings in the root xylem of perennial forbs, provides a valuable approach to detecting and assessing the effects of climate on herbaceous vegetation^24^,^25^,^26^,^27^. Recent studies have shown that the growth of herbaceous plants could be particularly sensitive and rapidly responsive to climatic fluctuation or extreme climatic events^24^,^25^, and could even be more sensitive to drought than tree growth due to probable lagged or prolonged effects in the latter^28^. Therefore, the application of herb-chronology may provide an opportunity to monitor, attribute and predict the dynamic response of herbaceous vegetation growth to climate change in semi-arid regions.

To control and alleviate soil erosion, some large-scale ecological restoration programs, particularly the ‘Grain-for-Green’ project (GFGP), have been launched since 1999 in the Loess Plateau^29^,^30^. According to the GFGP, introduced vegetation has become the main vegetation type^31^, playing an important role in decreasing soil erosion, carbon (C) and nitrogen (N) cycles^32^, and in reduce sediment transport into the Yellow River^33^. Many slope croplands have been converted into grasslands (including abandoned lands and pasture grasslands) in this region^34^. The grassland (including both native grassland and pasture grassland) is among the main types of vegetation, accounting for approximately one-third of the area in this region. This region has experienced rapid climate warming and increasing frequency and intensity of drought as a result of significantly increased temperature without corresponding increase in rainfall during the past five decades^35^. This makes it a potentially ideal region for examining the effects of temperature-induced drought stress on the growth of herbaceous vegetation. However, we know relatively little regarding the grassland response to climatic variations in this semi-arid region.

Thus, to better understand the dynamics of herbaceous vegetation in response to climate change, we used herb-chronology to investigate the growth of herbaceous plants in the Loess Plateau along a moisture gradient. In this region, alfalfa (*Medicago sativa*), as a preferred plant species to restore degraded ecosystems, is the main introduced species in pasture grassland, whereas Chinese cinquefoil (*Potentilla chinensis*) is one of the dominant perennial forbs in native grassland. The two species are perennial forbs and reliably represent the general conditions of the herbaceous vegetation due to their wide distribution in this region. We hypothesized that (1) increasing temperature, particularly in summer, has a significant negative effect on the growth of herbaceous plants due to a warming-induced water deficit, and (2) moisture plays a dominant role in regulating the growth of herbaceous plants in semi-arid areas of the Loess Plateau.

## Results

### Ring width patterns over time

**Figure 1:**
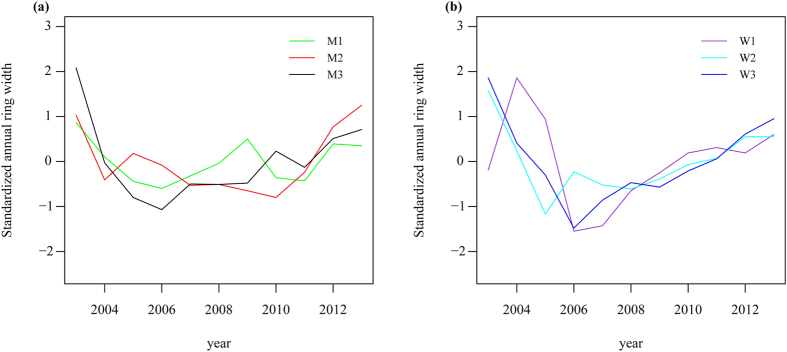
The detrended standardized ring widths (RW) of all samples of (**a**) *Medicago sativa* (M1, M2 and M3) and (**b**) *Potentilla chinensis* (W1, W2 and W3) from each site.

For both species and plots, the detrended standardized ring widths (RW) showed initially a highly consistent trend, beginning with a steep decreasing, followed by a gradual increasing in later years (Fig. 1a,b). The narrowest RW occurred in 2006 for both *M. sativa* (M1 and M3) and *P. chinensis* (M1 and M3). The average correlation coefficients between the individual RW series (also known as inter-series correlation) were 0.68, 0.85, 0.78, 0.99, 0.92 and 0.92 for sites M1, M2, M3, W1, W2 and W3, respectively, thus confirming the reliability for further analysis in this study.

### Relationships between RW and climate

**Figure 2:**
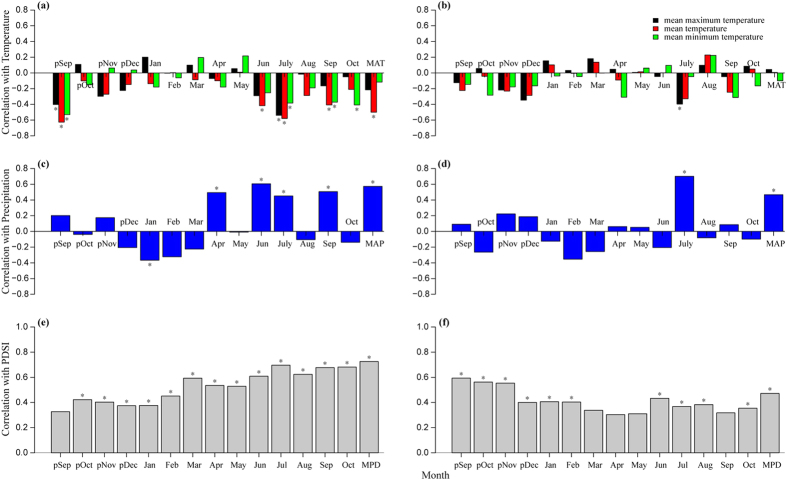
Pearson’s correlations between RW of *Medicago sativa* (left column) and *Potentilla chinensis* (right column), respectively, and the monthly mean temperature (**a**,**b**), total precipitation (**c,d**), and mean PDSI (**e,f**) from September of the previous year to October of the current year. Stars indicate a significant correlation (*P* < 0.05). MAP: mean annual precipitation, MAT: mean annual temperature, MPD: mean annual PDSI.

Results from the correlation analyses of between RW and each monthly climatic variable, including temperature variables (minimum, mean, maximum), precipitation and PDSI, are presented in Supplementary Figs S1, S2 and S3. All plots showed similar patterns for RW-temperature (see Supplementary Fig. S1), RW-precipitation (see Supplementary Fig. S2) and RW-PDSI (see Supplementary Fig. S3) correlations (*P* < 0.01). In our study region, the two species showed similar correlation patterns between RW and monthly climate factors (Fig. 2). For both species, the chronologies showed a significant positive correlation with PDSI from the previous September to the current year’s September (except the current year’s March, April, May and September in *P. chinensis*, *P* < 0.05, Fig. 2e,f) and precipitation in the current year’s July (*P* < 0.05, Fig. 2c,d) but a significant negative association with temperature in the current year’s July and October (*P* < 0.05, Fig. 2a,b).

**Figure 3:**
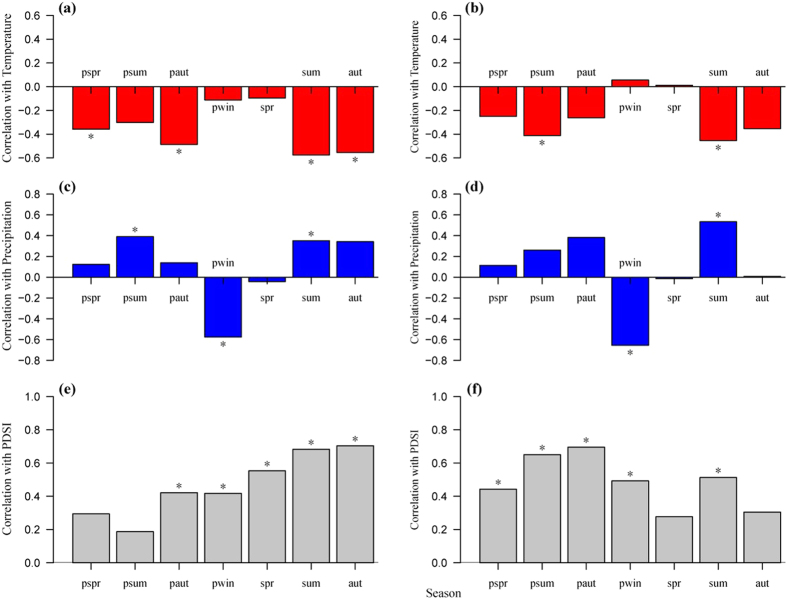
Pearson’s correlations between RW of *Medicago sativa* (left column) and *Potentilla chinensis* (right column), respectively, and seasonal temperature (**a,b**), total precipitation (**c,d**), and mean PDSI (**e,f**) from spring of the previous year to autumn of the current year. Stars indicate a significant correlation (*P* < 0.05). The seasons are previous spring (pspr), previous summer (psum), previous autumn (paut), previous winter (pwin), current year’s spring (spr), current year’s summer (sum) and current year’s autumn (aut).

The correlation analyses between RW and seasonal climatic variables performed for all six plots indicated similar patterns for RW-temperature (see Supplementary Fig. S4), RW-precipitation (see Supplementary Fig. S5) and RW-PDSI (see Supplementary Fig. S6) correlations (*P* < 0.01). For both species, RW showed a strong positive correlation with PDSI and a weaker, mostly positive correlation with precipitation but a negative association with temperature (Fig. 3). For *M. sativa*, RW showed a significant positive correlation with PDSI from the previous autumn to the current year’s autumn (P < 0.05, Fig. 3e), and with precipitation in the previous summer and current year’s summer (P < 0.05, Fig. 3c), but a significant negative association with temperature in the previous spring and autumn and the current year’s summer and autumn (P < 0.05, Fig. 3a). In addition, for *P. chinensis*, significant positive correlations were found in all seasons (except the current year’s spring and autumn) for PDSI (P < 0.05, Fig. 3f) and in the current year’s winter and previous summer for precipitation (P < 0.05, Fig. 3d), but a significant negative correlation was found in the previous summer and current year’s summer for temperature (P < 0.05, Fig. 3b).

All plots shared similar patterns in the relationships between RW and annual climatic factors independent from species. A significant positive correlations was found with annual total precipitation (*P* < 0.05, Fig. 2c,d) and mean PDSI (*P* < 0.05, Fig. 2e,f), while in contrast, a negative correlation was found with annual mean temperature (Fig. 2a,b).

At the species level, the RW of *M. sativa* showed generally stronger correlations with the considered climatic factors than *P. chinensis* (Figs 2 and 3). However, *P. chinensis* showed strong positive correlations with PDSI in the previous year, whereas *M. sativa* had relatively weak associations with PDSI in the previous year (Fig. 3).

## Discussion

In this study, herb-chronology data demonstrated that the growth of herbaceous plants in the Loess Plateau responded sensitively to monthly, seasonal and annual climate factors. The consistent pattern of strong correlations between RW and climate factors for both perennial forb species in all plots implies that the growth of herbaceous plants in the study region was highly controlled by climate conditions during the previous and current year’s growing season.

The two species showed significant positive correlations with precipitation and PDSI but a significant negative correlation with temperature in summer (Fig. 3), particularly in July (Fig. 2), which is consistent with recent studies based on large-scale satellite observations^12^,^17^. China has experienced a remarkable greening trend over the last three decades^17^, particularly in the Loess Plateau where the net significant vegetation greening is mostly due to ecological restoration programs^12^. It has been suggested that climate warming has a significant negative effect on vegetation greening, particularly during the growing season, and even leads to vegetation browning^12^. Furthermore, significant warming has resulted in a decreasing Leaf Area Index in northern China^17^ and driven tree growth decline in the semi-arid forests of Inner Asia^7^. Low PDSI during the growing season, particularly in summer (see Supplementary Fig. S7), indicates water limitation, which is the result of temperature-driven high evaporation rates that are not fully compensated by the concomitant higher precipitation in this region. As a consequence, the temperature-induced water deficit during the growing season inhibited the growth of herbaceous plants by decreasing photosynthetic activity, nitrogen and carbon productivity^36^,^37^. In addition, the mean annual temperature has significantly increased over the last 50 years, whereas the total annual precipitation has undergone non-significant decline in this region^35^. While vegetation transpiration and soil evaporation can consume 90% of total precipitation^38^, and continuously rising temperature increased transpiration and evaporation, and thus likely exacerbated low soil moisture content in this region. In this context, our results indicate that rising temperature in a semi-arid area, particularly in summer, has a strong negative impact on the growth of herbaceous vegetation. In contrast and supporting the aforementioned explanation, the association with spring temperature was markedly weaker (Fig. 3), suggesting that increasing temperature at the beginning of the growing season only weakly affects the growth of herbaceous plants, presumably due to lower evapotranspiration in our semi-arid locations.

In this study, we also analyzed the effects of climate variations in the previous year on the growth of herbaceous plants. Recent studies based on tree-ring data have shown that climatic variables in the previous year have a strong influence on tree^37^,^39^,^40^ or shrub growth^41^. We found that all species showed a strong positive correlation with precipitation and PDSI but a negative correlation with temperature in the summer and autumn of the calendar year prior to the growing season (Fig. 3). These findings indicated that drought stress driven by increasing temperature in the previous year could continue to limit the growth of herbaceous plants in the subsequent year in semi-arid sites. While this could point to a limited pool of stored resources that carries over to the following year, it could also be simply explained by a reduction in soil water availability that persist until the following growing season, particularly when considering the relatively dry spring conditions^42^. However, both species exhibited significant negative correlations with precipitation in previous winter (Fig. 3). This phenomenon has also been previously observed in tree ring research^43^,^44^.

It has been widely reported that PDSI, combined with the influence of temperature and precipitation, better reflect the variation of moisture and drought in an arid or semi-arid area^40^,^45^,^46^. Our results demonstrated that the two species showed significant positive correlations with the PDSI data not only in the current year, but also in the previous year (Figs 2 and 3), which is consistent with previous results based on tree rings in northwest China forests^37^. The stronger correlations between RW and PDSI confirmed that moisture played a major role in regulating the growth of herbaceous plants in these semi-arid sites.

Additionally, several studies have reported that introduced species have relatively higher stomatal conductance, growth rates and water use than native species^47^,^48^,^49^,^50^, which may lead to excessive depletion of soil water under generally dry conditions. Evidence based on field observation suggests that compared to the soil moisture of native grassland, *M. sativa* pasture grassland can drastically deplete deep soil moisture in semi-arid regions of the Loess Plateau^51^,^52^,^53^. Indeed, since *M. sativa* was planted in this region, soil water significantly decreased, even to the point of desiccation in deeper soil layers^54^. In addition, the high planting density of the introduced pasture can contribute to the soil moisture deficit^53^. Pasture grassland shows lower water availability than native grassland due to the excessive depletion of soil moisture. Thus, the relatively high soil moisture content in native grassland in the previous year could alleviate water deficit and benefit to plant growth during the subsequent growing season, particularly in a drought year. This phenomenon may help to explain why *P. chinensis* showed a strong positive correlation with PDSI in the previous year, while *M. sativa* was relatively weakly associated with PDSI in the previous year (Fig. 3). Furthermore, it has been suggested that climate warming has a stronger negative effect on introduced species in regions where water availability is lower due to warming-induced increased evapotranspiration^50^,^55^,^56^. In fact, evidence from a field warming experiment in a native lowland grassland in the Australia indicated that rising temperature resulted in a significant reduction of population growth rate for introduced species, whereas population growth rate of native species were not significantly affected^55^. This phenomenon may help to explain why *M. sativa* was more sensitive to climatic factors than *P. chinensis* in our semi-arid sites examined (Figs 2 and 3).

In summary, to our knowledge, this study is the first to show by the method of herb-chronology, that climate warming has a significant negative impact on the growth of herbaceous plants due to water deficit associated with increasing temperature in a semi-arid area in the Loess Plateau of China. This study revealed that climate warming may have stronger influences on introduced pasture grassland than native grassland. In addition, our results showed that herb-chronology is a very useful approach for detecting the growth dynamics of herbaceous plants and evaluating the relationship between herbaceous vegetation and climate change. Hence, since a majority of climate models project an increasing warming and drying trend for the Loess Plateau in the future, more appropriate native species should be carefully selected and implemented in ecological restoration programs to promote sustainable ecosystems in order to mitigate negative effects of climatic change.

## Materials and Methods

### Study area and sampling sites

**Figure 4:**
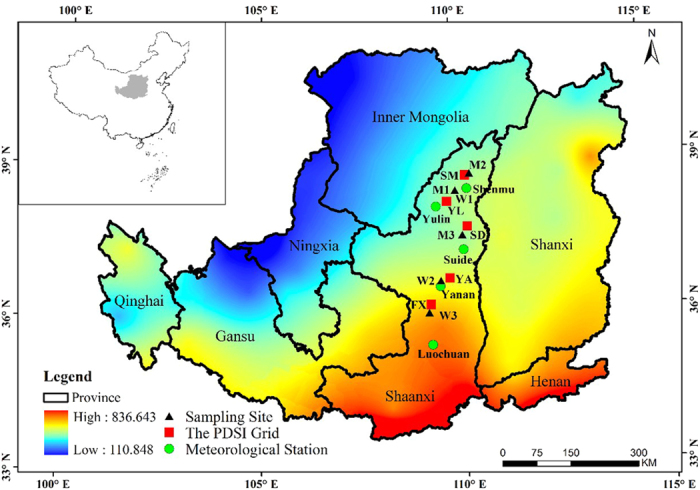
Map showing the six sampling sites (black triangles), the nearest five meteorological stations (green circles) from Chinese meteorological stations, and the nearest six PDSI grids (red squares) from the KNMI Climate Explorer at 0.5° spatial resolution. The both maps were created using ESRI ArcGIS 9.3 (http://www.esri.com/).

The study area is located in the Central Loess Plateau in the northern Shaanxi Province, China (Fig. 4), which is characterized by deep loess deposits and by rolling and hilly topography. The region is dominated by a temperate, semi-arid continental monsoon climate. The mean annual temperature ranges from 10.8 to 9.3 °C, and the annual total precipitation ranges from 607 to 424 mm from the south to the north (according to data from Chinese meteorological stations from 2002 to 2013). More than seventy percent of the annual total precipitation falls from June to September, while the mean annual temperature is highest in July (see Supplementary Fig. S7).

In 2014, six plots (three per species) were selected. *M. sativa* plots (M1, M2 and M3) were located in pasture grassland, whereas *P. chinensis* plots (W1, W2 and W3) were located in native grassland (Fig. 4, Table 1). All plots featured similar hill slopes (approximately 15–25°) and were carefully chosen to avoid disturbance such as grazing, irrigation, or human activity.

### Herb-chronology sampling and laboratory processing

A total of 90 generally larger *M. sativa* and *P. chinensis* plants (15 per plot) were carefully sampled avoiding unhealthy individuals and individuals showing substantial root decay. Taproots of plants were carefully excavated to a soil depth of approximately 10 cm (thus including the root collar) and kept frozen to preserve before laboratory processing.

**Figure 5:**
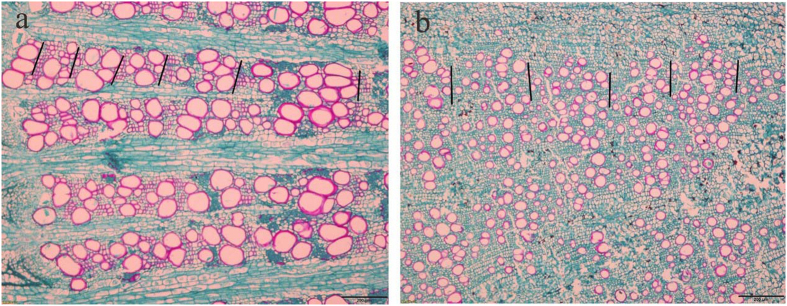
Distinct growth rings in the secondary xylem of the root collar of (**a**) *Medicago sativa* and (**b**) *Potentilla chinensis* as viewed through a microscope. Scale bars = 200 μm.

In the laboratory, approximately l cm long segments were cut from the root collar with a razor blade. Then, approximately 10–15 μm thick cross-sections were produced using a sledge microtome^57^. The cross-sections were stained with safranin and fast green solution to distinguish between lignified (red) and non-lignified (green) xylem cell structures (Fig. 5). Digital images of root cross sections were captured by using a digital camera (OLYMPUS, DP26-CU, resolution 2448 × 1920 pixels). Annual growth rings were analyzed by recognizing earlywood (large vessels) and latewood (small vessels) in the secondary root xylem^58^,^59^ (Fig. 5), and plant age was estimated by counting the number of annual rings^27^. The age of *M. sativa* plants ranged from 11–13 years (the number of 13-year-old individuals for each site was over 10), while the age of *P. chinensis* plants was in the range of 9 to 13 years (most samples were more than 11 years old). Because the samples were not collected at the end of the growing season, the growth ring for 2014 was excluded from our analysis. Similarly, the growth ring of the innermost year was also excluded since it was often partly decayed^58^,^60^. The ring widths from three separate representative radii per individual were measured manually using cellSens software (OLYMPUS), and the average of the three measurements was used as a proxy for the width of the respective annual ring.

### Climate data

The meteorological data used in this study included monthly mean temperature, monthly minimum temperature, monthly maximum temperature and total monthly precipitation for the period 2002–2013, and were obtained from the nearest meteorological stations, i.e. Shenmu, Yulin, Suide, Yanan and Luochuan (Fig. 4, Table 1). The monthly Palmer drought severity index (PDSI) data were obtained from the closest grid point (0.5° geographic resolution) of the KNMI Climate Explorer (http://climexp.knmi.nl/) for the period from 2002 to 2012 (Fig. 4, Table 1). Analyses of seasonal climate were conducted for a 21-month time windows, from March in the previous year to November in the current year. Seasonal (spring: March-May, summer: June-August, autumn: September-November, and winter: December-February) mean temperature, total precipitation and PDSI were calculated according to the monthly climate data. The winter in the current year was removed because the forbs had already stopped growing by then.

### Statistical analysis

For both species, standardized ring widths showed a significant (*P* < 0.01) decreasing trend with increasing age at all sites except M2 (see Supplementary Fig. S8), indicating that obvious growth trends existed in most individuals. It has been suggested that plant age causes this general declining trend, whereas climate is responsible for the superimposed year to year variability^26^. It was therefore necessary to select an appropriate approach to remove the age-related growth in order to analyze the targeted relationship between plant growth and climate.

Detrending methods using linear regression lines or negative exponential curves are common ways to remove biological growth trends in tree-ring chronology^61^. To find a suitable detrending method, we performed a test comparing the two aforementioned methods to remove the growth trends of individuals for sites M3, W1, respectively. We found that the ring widths series detrended using a linear regression line was significantly correlated with the one detrended using a negative exponential curve for sites M3 (*r* = 0.92, *p* < 0.01), W1 (*r* = 0.91, *p* < 0.01), respectively. The two approaches shared similar patterns of correlation with climate (see Supplementary Fig. S9). Because both forb species only had relatively short ages, we therefore considered detrending using linear regression an appropriate method for this study, which was a relatively simple and conservative detrending method that can retain climate signal.

Furthermore, we tested the relationship between absolute growth (standardized ring widths) and climate using individuals at site M1. The residual growth (detrended standardized ring widths) showed stronger correlations with monthly, seasonal and annual climate variables (temperature, precipitation and PDSI) than the absolute growth (see Supplementary Figs S10 and S11). Thus, it is reasonable to analyze the correlation between residual growth and climate. After these initial tests, the ring widths values of all individuals for all plots (except M2) were detrended with linear regression lines and each plot-level series (including M2) was standardized to zero mean and unit standard deviation^24^,^25^. The detrended standardized ring widths (RW) for each plot were used to relate year-to-year variability in forb growth to climate.

The forb growth-climate relationships were analyzed over the period 2003–2013. Pearson’s correlations were calculated for each plot between RW and monthly, seasonal and annual climatic factors (see Supplementary Figs S1, S2, S3, S4, S5 and S6). RW was correlated with monthly temperature, precipitation and PDSI, respectively, from the previous September to the current year’s October to identify which monthly climate factors affected plant growth. Additionally, RW was also correlated with seasonal temperature, precipitation and PDSI, respectively, from the previous spring to the current year’s autumn to identify which seasonal climate factors influenced the growth of forbs.

All analyses were conducted with R 3.1.0 (R Development Core Team).

## Additional Information

**How to cite this article**: Shi, S. *et al.* Roots of forbs sense climate fluctuations in the semi-arid Loess Plateau: Herb-chronology based analysis. *Sci. Rep.* **6**, 28435; doi: 10.1038/srep28435 (2016).

## Supplementary Material

Supplementary Information

## Figures and Tables

**Table 1:** Statistics of the six sampling sites, five meteorological stations and PDSI grids from the KNMI Climate Explorer at 0.5° spatial resolution.

Data type	Site code	Location (latitude N; longitude E)	Elevation (m)	Number of samples
*Medicago sativa*	M1	38.46°, 109.98°	1328	15
	M2	38.79°, 110.37°	1240	15
	M3	37.59°, 110.11°	1058	15
*Potentilla chinensis*	W1	38.46°, 109.98°	1328	15
	W2	36.71°, 109.52°	1201	15
	W3	36.09°, 109.2°	1275	15
Meteorological data	Shenmu	38.49, 110.28	1098	–
	Yulin	38.16°, 109.47°	1157	–
	Suide	37.3°, 110.13°	930	–
	Yanan	36.6°, 109.5°	959	–
	Luochuan	35.46°, 109.25°	1156	–
PDSI	SM	38.75°, 110.25°	–	–
	YL	38.25°, 109.75°	–	–
	SD	37.75°, 110.25°	–	–
	YA	36.75°, 109.75°	–	–
	FX	36.25°, 109.25°	–	–
