# Supplementary material for: Roots of forbs sense climate fluctuations in the semi-arid Loess Plateau: Herb-chronology based analysis
Source: Sci Rep. 2016 Jun 21;6:28435. doi: 10.1038/srep28435 (PMC4914992; doi:10.1038/srep28435)
Supplement: Supplementary Information [file srep28435-s1.pdf]

Supplementary information

Roots of forbs sense climate fluctuations in the semi-arid Loess Plateau: Herb-chronology based analysis

Songlin Shi, Zongshan Li, Hao Wang, Georg von Arx, Yihe Lü, Xing Wu, Xiaochun Wang, Guohua Liu and Bojie Fu

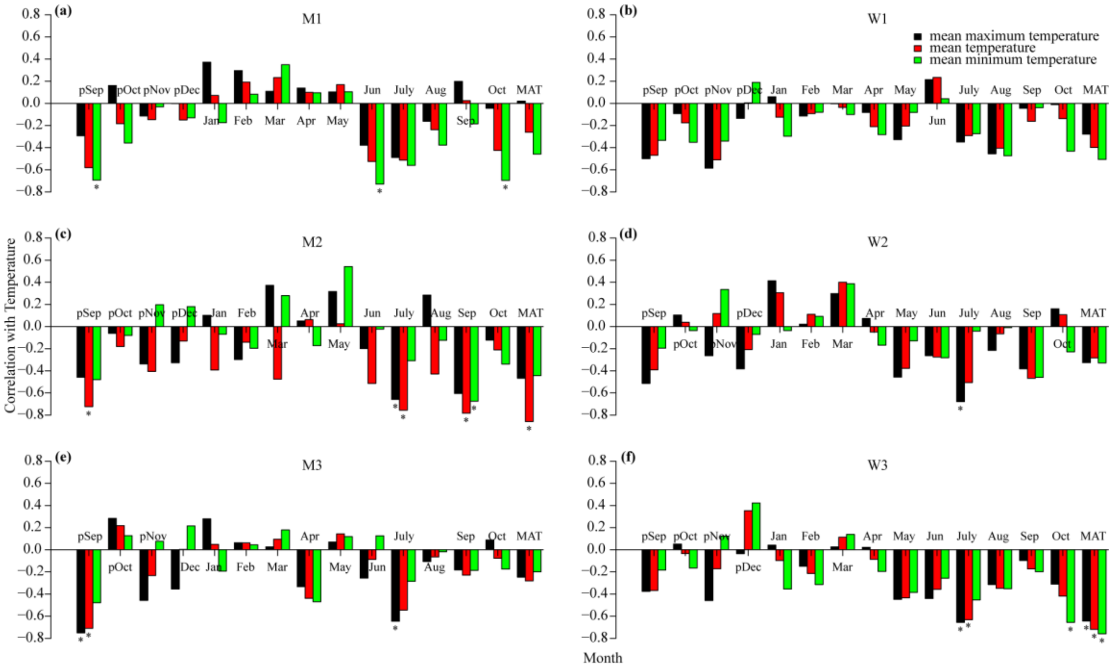

Figure S1. Pearson's correlations between the detrended standardized ring widths (RW) of *Medicago sativa* (left column) and *Potentilla chinensis* (right column), respectively, and the monthly mean temperature from September of the previous year to October of the current year at each site. Stars indicate a significant correlation (P<0.05), MAT: mean annual temperature.

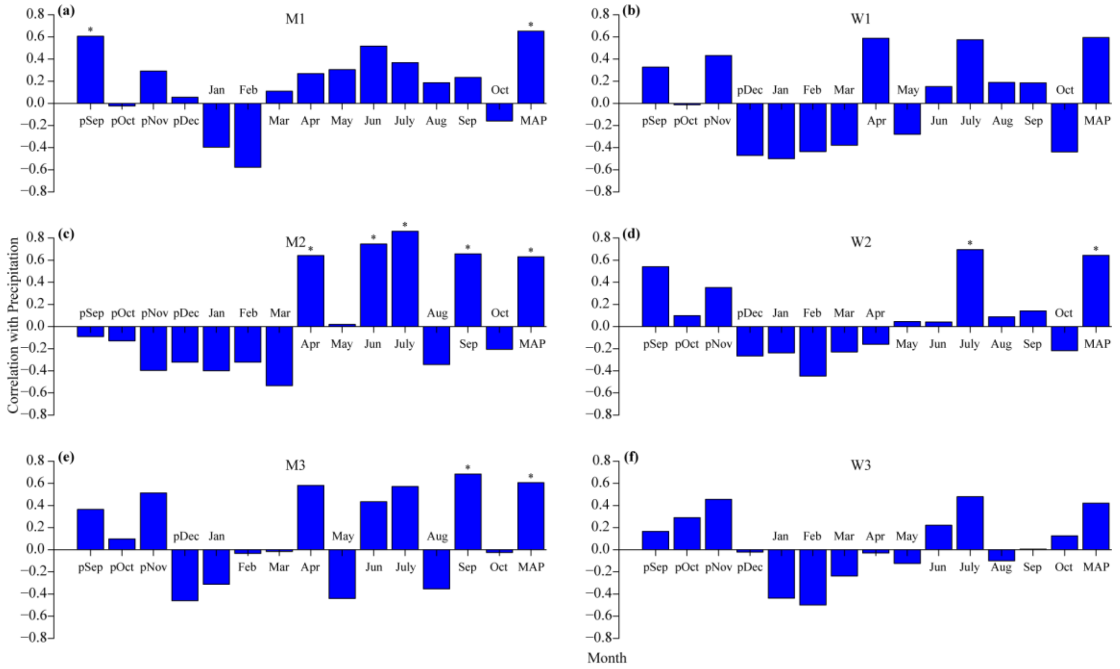

Figure S2. Pearson’s correlations between the RW of *Medicago sativa* (left column) and *Potentilla chinensis* (right column), respectively, and the monthly total precipitation from September of the previous year to October of the current year in each plot. Stars indicate a significant correlation (*P*<0.05), MAP: annual total precipitation.

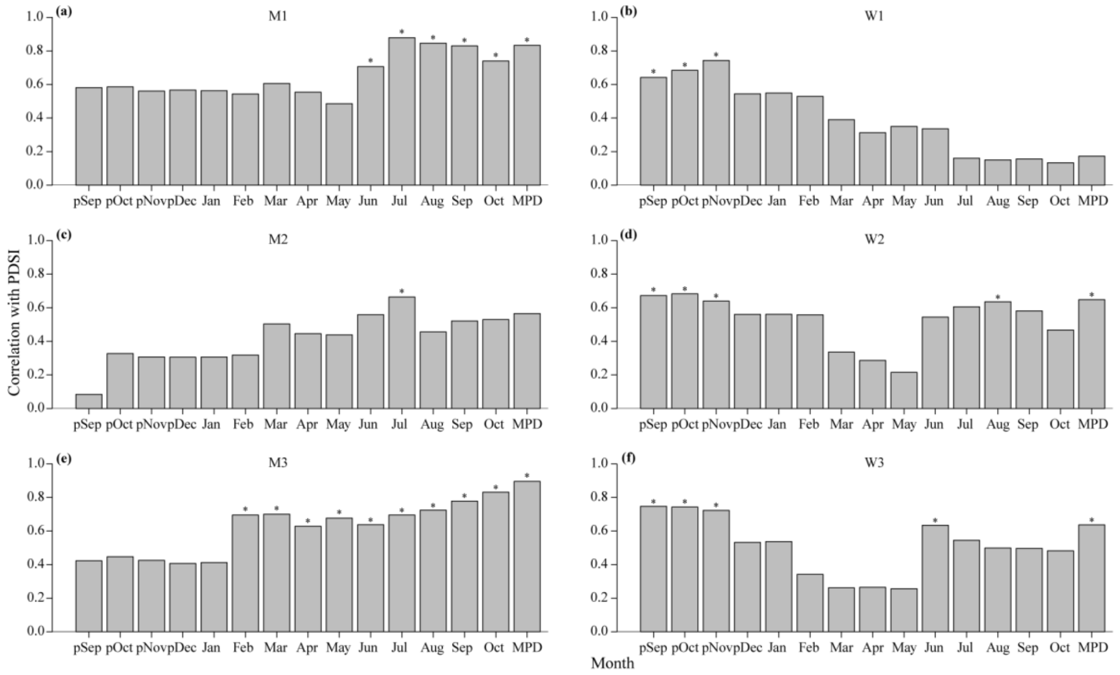

Figure S3. Pearson's correlations between the RW of *Medicago sativa* (left column) and *Potentilla chinensis* (right column), respectively, and the monthly mean PDSI from September of the previous year to October of the current year in each plot. Stars indicate a significant correlation ($P<0.05$), MPD: mean annual PDSI.

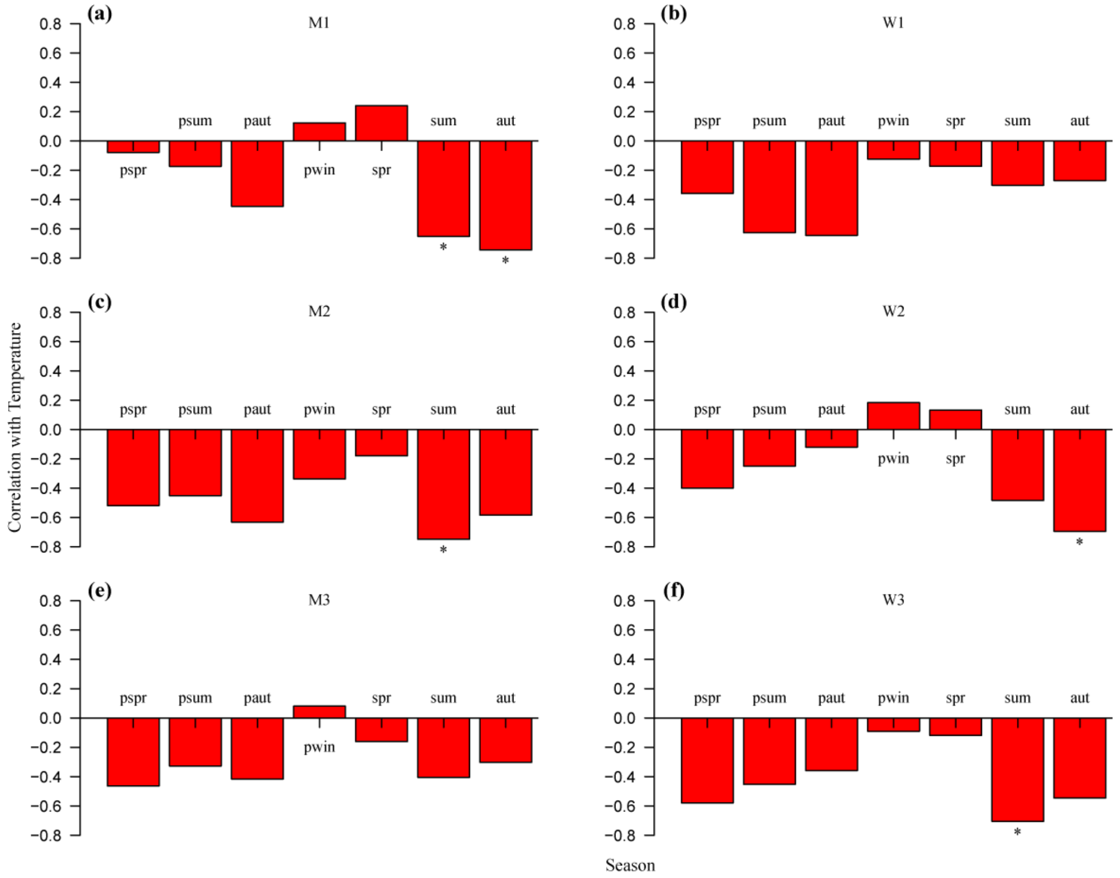

Figure S4. Pearson's correlations between the RW of *Medicago sativa* (left column) and *Potentilla chinensis* (right column), respectively, and seasonal temperature from spring of the previous year to autumn of the current year in each plot. Stars indicate a significant correlation (P<0.05). The seasons are previous spring (pspr), previous summer (psum), previous autumn (paut), previous winter (pwin), current year's spring (spr), current year's summer (sum) and current year's autumn (aut).

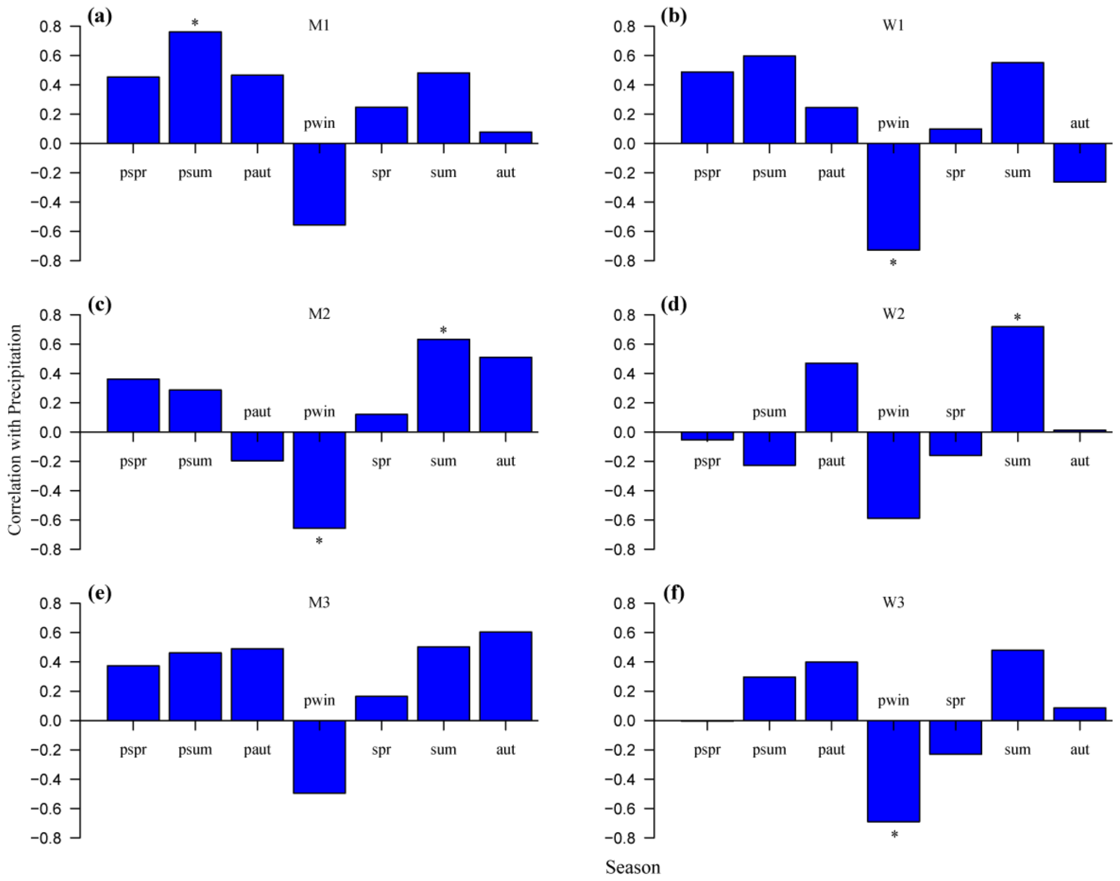

Figure S5. Pearson's correlations between the RW of *Medicago sativa* (left column) and *Potentilla chinensis* (right column), respectively, and seasonal precipitation from spring of the previous year to autumn of the current year in each plot. Stars indicate a significant correlation (P<0.05). The seasons are previous spring (pspr), previous summer (psum), previous autumn (paut), previous winter (pwin), current year's spring (spr), current year's summer (sum) and current year's autumn (aut).

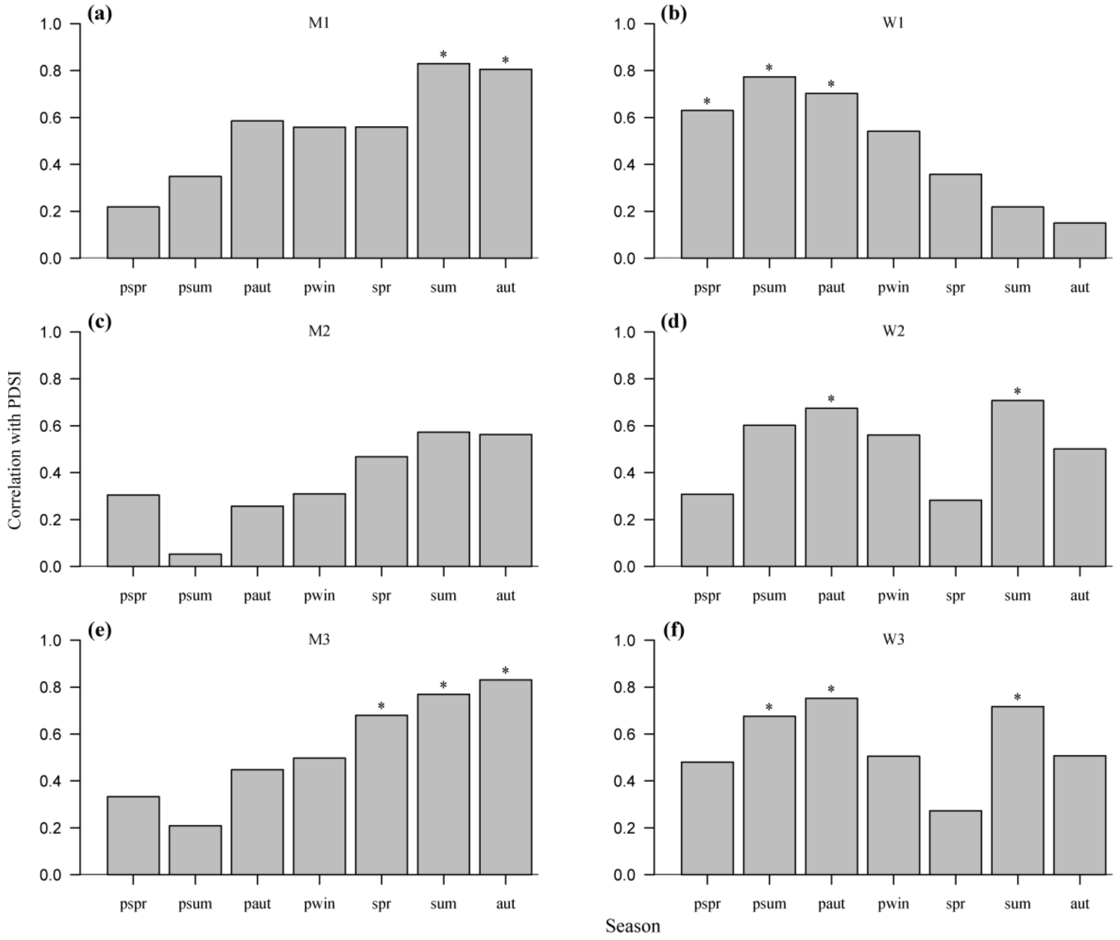

Figure S6. Pearson's correlations between the RW of Medicago sativa (left column) and Potentilla chinensis (right column), respectively, and seasonal PDSI from spring of the previous year to autumn of the current year in each plot. Stars indicate correlation that is significant (P<0.05). Stars indicate a significant correlation (*P*<0.05). The seasons are previous spring (pspr), previous summer (psum), previous autumn (paut), previous winter (pwin), current year's spring (spr), current year's summer (sum) and current year's autumn (aut).

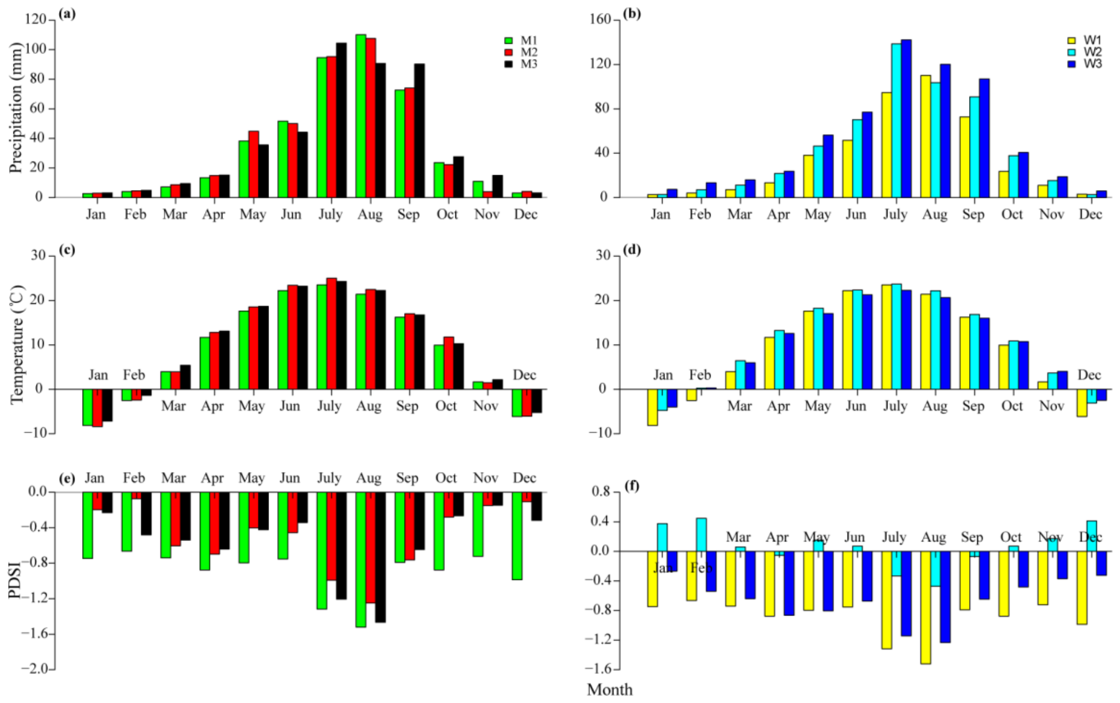

Figure S7. Monthly total precipitation (a, b) and monthly mean temperature (c, d) records from the meteorological stations and monthly mean PDSI (e, f) from the KNMI Climate Explorer during 2002- 2013 at the six sampling sites.

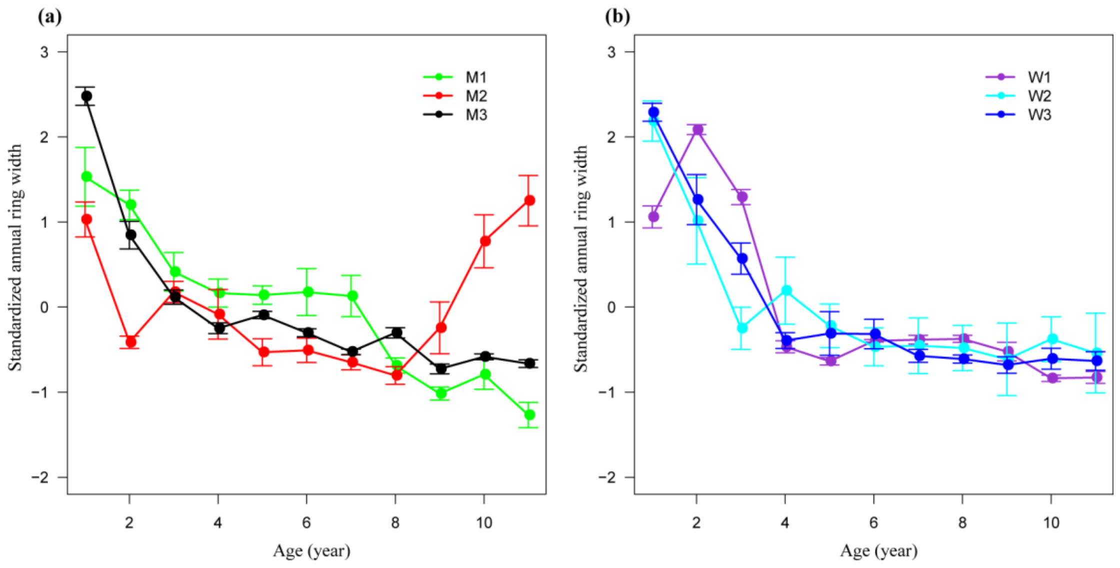

Figure S8. Standardized ring widths trend with increasing age for (a) *Medicago sativa* (M1, M2 and M3) and (b) *Potentilla chinensis* (W1, W2 and W3) at each site.

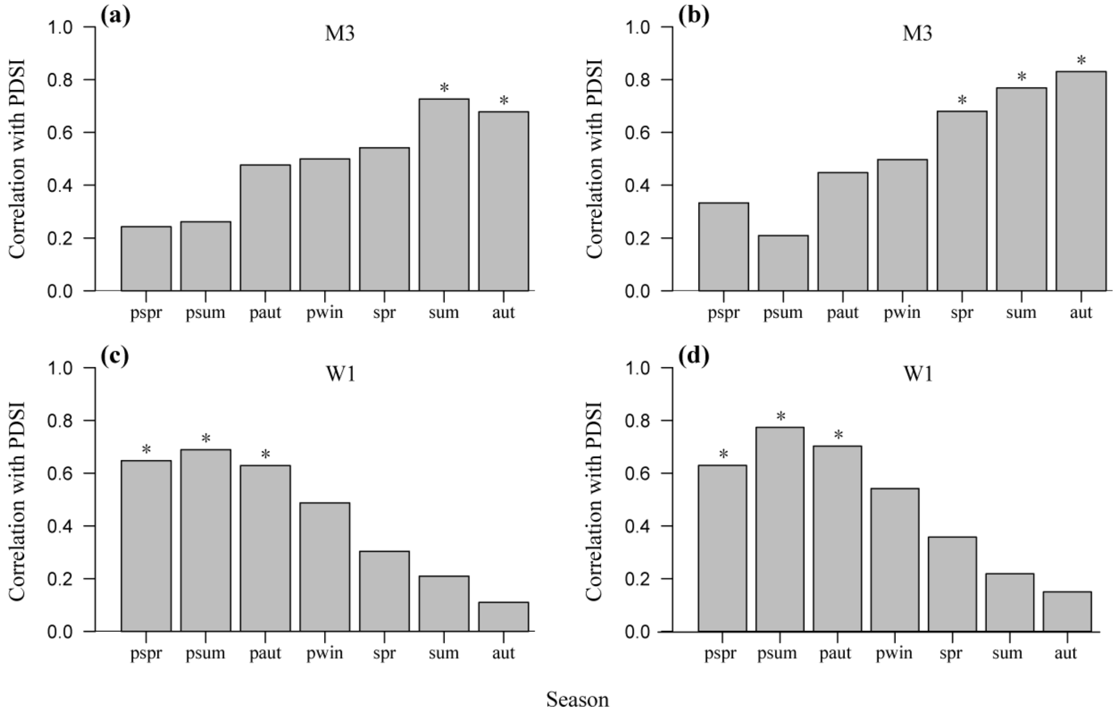

Figure S9. Pearson's correlations between seasonal PDSI and ring widths detrended with negative exponential curves (a, c) and linear regression lines (b, d) for *Medicago sativa* at site M3, W1, respectively. Stars indicate a significant correlation (P<0.05). The seasons are previous spring (pspr), previous summer (psum), previous autumn (paut), previous winter (pwin), current year's spring (spr), current year's summer (sum) and current year's autumn (aut).

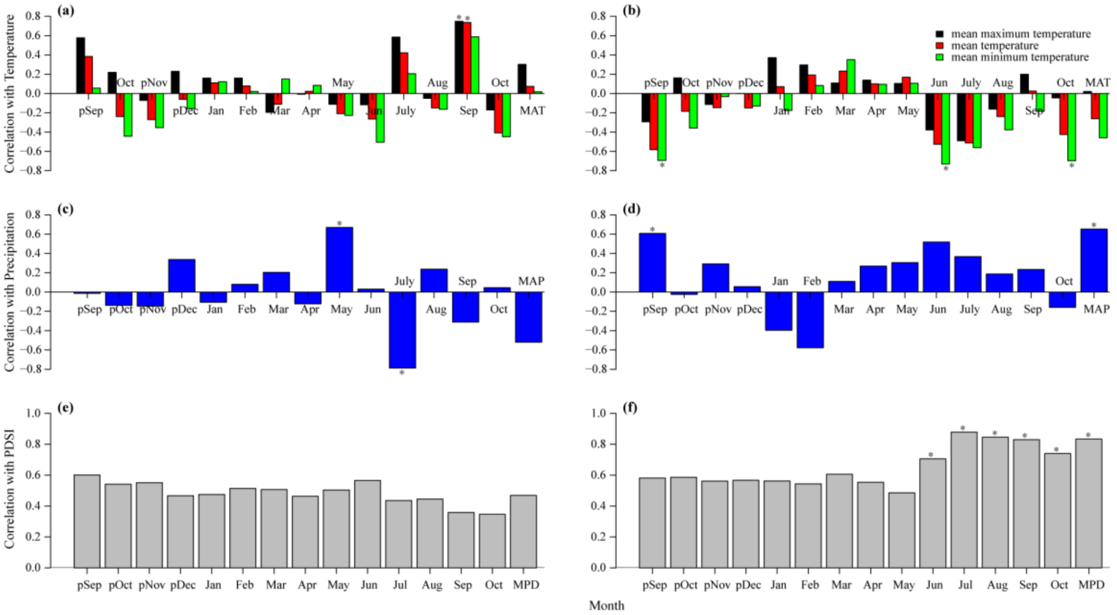

Figure S10. Pearson's correlations between the absolute growth (standardized ring widths, left column), the residual growth (detrended standardized ring widths, right column) of *Medicago sativa* at site M1, and the monthly mean temperature (a, b), total precipitation (c, d) and PDSI (e, f) from September of the previous year to October of the current year. Stars indicate a significant correlation (P<0.05). MAP: annual total precipitation, MAT: mean annual temperature, MPD: mean annual PDSI.

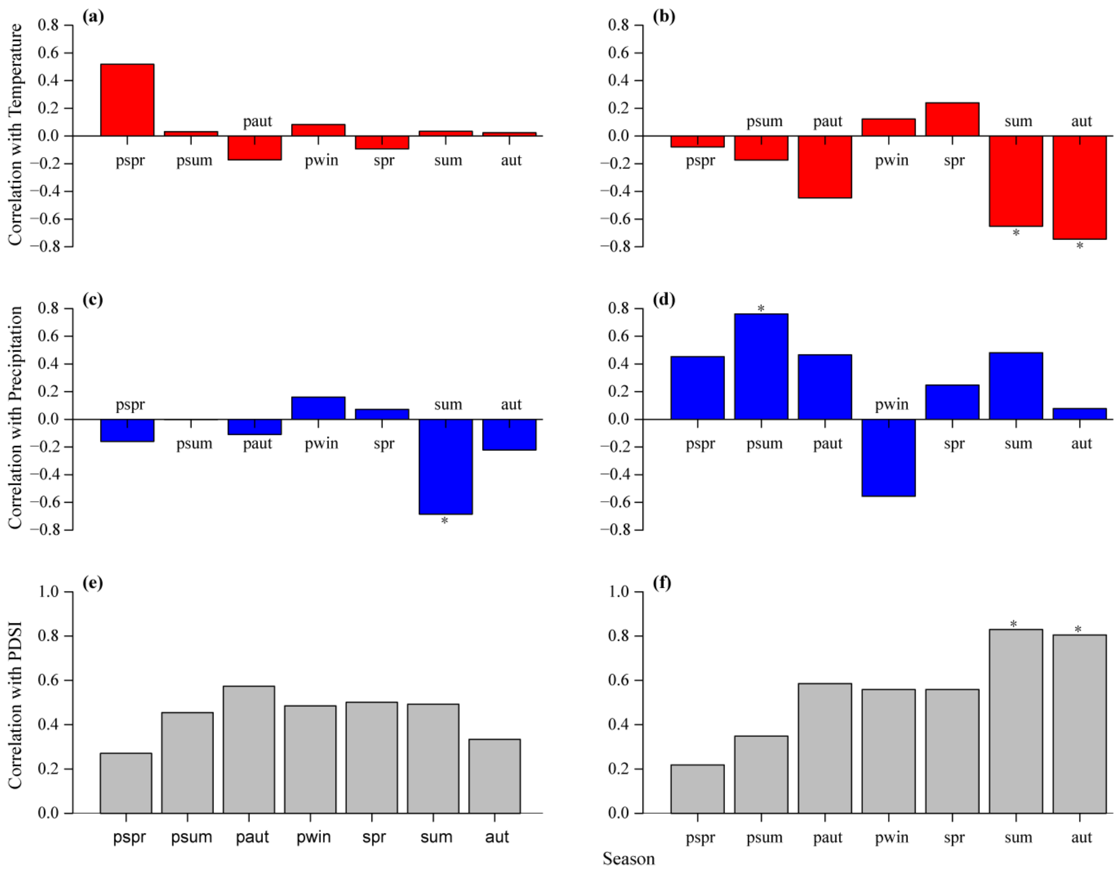

Figure S11. Pearson's correlations between the absolute growth (standardized ring widths, left column), the residual growth (detrended standardized ring widths, right column) of *Medicago sativa* at site M1, and seasonal temperature (a, b), total precipitation (c, d) and PDSI (e, f) from spring of the previous year to autumn of the current year. Stars indicate a significant correlation (P<0.05). The seasons are previous spring (pspr), previous summer (psum), previous autumn (paut), previous winter (pwin), current year's spring (spr), current year's summer (sum) and current year's autumn (aut).
